# Gonioscopy-assisted transluminal trabeculotomy for open-angle glaucoma with failed incisional glaucoma surgery: two-year results

**DOI:** 10.1186/s12886-023-02830-7

**Published:** 2023-03-06

**Authors:** Yiwei Wang, Weijia Zhang, Chen Xin, Jinghong Sang, Yang Sun, Huaizhou Wang

**Affiliations:** 1 Department of Ophthalmology, Henan Provincial People’s Hospital, People’s Hospital of Zhengzhou University, Zhengzhou, 450003 China; 2 Beijing Institute of Ophthalmology, Beijing Tongren Hospital, Capital Medical University, Beijing, 100730 China; 3 Department of Ophthalmology, Peking University Third Hospital, Beijing, 100191 China; 4 Beijing Key Laboratory of Restoration of Damaged Ocular Nerve, Peking University Third Hospital, Beijing, 100191 China; 5 Department of Ophthalmology, Beijing Tongren Hospital, Capital Medical University, No.8 Chongwenmennei Street, Beijing, 100730 Dongcheng District China; 6 Department of Ophthalmology, Stanford University, Palo Alto, CA 94303 USA

**Keywords:** Gonioscopy-assisted transluminal trabeculotomy, GATT, Minimally invasive glaucoma surgery, Primary open-angle glaucoma, Juvenile onset open-angle glaucoma, Refractory glaucoma, Treatment

## Abstract

**Background:**

To evaluate the safety and efficacy of gonioscopy-assisted transluminal trabeculotomy (GATT) in treating patients with open-angle glaucoma (OAG) who had failed prior incisional glaucoma surgery.

**Methods:**

A consecutive case series of OAG patients aged ≥ 18 who underwent GATT with previous failed glaucoma incision surgery was retrospectively analyzed. Main outcome measures included intraocular pressure (IOP), the number of glaucoma medications, surgical success rate, and occurrence of complications. Success was defined as an IOP of ≤ 21 mmHg and a reduction of IOP by 20% or more from baseline with (qualified success) or without (complete success) glaucoma medications. For eyes with preoperative IOP of < 21 mmHg on 3 or 4 glaucoma medications, postoperative IOP of ≤ 18 mmHg without any glaucoma medications was also defined as complete success.

**Results:**

Forty-four eyes of 35 patients (21 with juvenile-onset open-angle glaucoma and 14 with adult-onset primary open-angle glaucoma) with a median age of 38 years were included in this study. The proportion of eyes with 1 prior incisional glaucoma surgery was 79.5%, and the others had 2 prior surgeries. IOP decreased from 27.4 ± 8.8 mm Hg on 3.6 ± 0.7 medications preoperatively to 15.3 ± 2.7 mm Hg on 0.5 ± 0.9 medications at the 24-month visit (*P* < 0.001). The mean IOP and the number of glaucoma medications at each follow-up visit were lower than the baseline (all *P* < 0.001). At 24 months postoperatively, 82.1% of the eyes had IOP ≤ 18 mmHg (versus 15.9% preoperatively, *P* < 0.001), 56.4% reached IOP ≤ 15 mmHg (versus 4.6% preoperatively, *P* < 0.001), and 15.4% achieved IOP ≤ 12 mmHg (compared to none preoperatively, *P* = 0.009). While 95.5% of eyes took 3 or more medications preoperatively, 66.7% did not take glaucoma medication 24 months after GATT. Thirty-four (77.3%) eyes achieved IOP reduction greater than 20% on fewer medications. The complete and qualified success rates were 60.9% and 84.1%, respectively. No vision-threatening complications occurred.

**Conclusions:**

GATT was safe and effective in treating refractory OAG patients who failed prior incisional glaucoma surgery.

## Introduction

Glaucoma is the leading cause of irreversible blindness and estimated to affect approximately 111.8 million individuals worldwide by 2040 [1]. A systematic analysis revealed that the overall prevalence of primary open-angle glaucoma (POAG) in China was 1.02% in 2015 [2]. Glaucoma filtering surgery is one of the most frequently performed anti-glaucoma procedures in China [3]. However, bleb failure due to episcleral fibrosis or subconjunctival scarring has always been a challenge, even with the introduction of releasable sutures and the combined use of antimetabolites. Bleb-independent surgeries with more favorable safety profile are needed to preserve vision in glaucoma patients.

Gonioscopy-assisted transluminal trabeculotomy (GATT), a bleb-free procedure, creates a direct communication between aqueous humor and collector channels by circumferentially opening trabecular meshwork and inner wall of Schlemm’s canal, and consequently facilitates aqueous drainage [4]. Previous studies have shown that GATT can effectively treat open-angle glaucoma (OAG) [5–13] and childhood glaucoma [14–19].

Patients with prior failed glaucoma surgery are more prone to bleb fibrosis after repeated filtering surgery. GATT may show promise for these cases. Grover DS et al. [20] reported for the first time that GATT alone or combined with cataract surgery was safe and successful in treating 60% to 70% of open-angle glaucoma with prior incisional glaucoma surgery. Sarkisian SR et al. [21] evaluated outcomes of 360° ab-interno trabeculotomy using the TRAB360 device and confirmed the work by Grover DS and colleagues. By contrast, Cubuk MO et al. [22] reported a limited efficacy of GATT for treating POAG patients with failed trabeculectomy. Thus, further research is required to evaluate this procedure.

The current study aimed to evaluate the efficacy and safety of GATT for treating OAG patients with prior failed incisional glaucoma surgery.

## Methods

### Participants and design

This consecutive case series included OAG patients aged ≥ 18, who previously had undergone incisional glaucoma surgery and presented an IOP > 21 mm Hg or progressive deterioration of visual field defects on maximum medication, or intolerance to medical treatment, and consequently underwent GATT in Beijing Tongren Hospital between May 2018 and March 2019. Exclusion criteria included primary congenital glaucoma, secondary open-angle glaucoma or chronic angle-closure glaucoma, bleeding disorders, or inability to stop anticoagulant medications. All patients had a preoperative gonioscopic examination. Staging of glaucoma severity was according to Hodapp-Parrish-Anderson criteria of visual field defect [23]. Written informed consent was obtained from all patients before surgery. This study followed the tenants of the Declaration of Helsinki and was approved by the institutional ethics committee at Beijing Tongren Hospital.

### Surgical procedure and post-operative care

All cases underwent GATT surgery as a stand-alone procedure performed by Dr. Huaizhou Wang. The surgical technique has previously described [4]. Briefly, a clear corneal temporal incision was created and viscoelastic material was injected into the anterior chamber. A microcatheter was inserted into the anterior chamber through a corneal paracentesis track placed in the superonasal or inferonasal quadrant. Using direct visualization with a goniolens, a 1–2 mm goniotomy was created in the nasal angle. Microsurgical forceps were placed into anterior chamber through the temporal clear corneal incision and used to insert the iTrack illuminated microcatheter (Ellex iScience Inc., Fremont, CA) into Schlemm’s canal through the goniotomy incision and advance the catheter through the canal circumferentially. Circumferential trabeculotomy was performed by pulling both ends of the microcatheter. If the microcatheter stopped somewhere in the Schlemm’s canal, an ab-interno cut down was performed to achieve a partial trabeculotomy, and the microcatheter was re-inserted into the original goniotomy incision in the opposite direction to achieve as close to 360-degree trabeculotomy as possible. The viscoelastic was then removed from the anterior chamber. The corneal wounds were hydrated to ensure watertight closure.

After surgery, all the subjects were given tobramycin-dexamethasone (TobraDex, Alcon, Rijksweg, Belgium) and Pranoprofen (Pranopulin, Senju Pharmaceutical, Osaka, Japan) eyedrops for 2 to 4 weeks. Pilocarpine 2% (Bausch & Lomb, Rochester, NY, USA) was used 4 times daily for 3 months to hinder peripheral anterior synechia and therefore not considered as an anti-glaucoma medication during this time.

### Main outcome measurements

Demographics, baseline clinical characteristics, and postoperative data were obtained from patients' medical records at each visit. The postoperative examinations were performed on day 1, 2, 3 and 7, week 2 to 3, month 1, 3, 6, and thereafter every 3 to 6 months. Surgical success was defined as IOP ≤ 21 mmHg with at least a 20% reduction from baseline with (qualified success) or without (complete success) the use of glaucoma medications. Individuals with postoperative IOP of ≤ 18 mmHg without any glaucoma medications, if preoperative IOP of ≤ 21 mmHg on at least 3 glaucoma medications, were also defined as complete success. Eyes with visual acuity deteriorated to an absence of light perception were defined as a surgical failure; those undergoing a secondary surgical procedure were also defined as a failure and excluded from analysis.

### Statistical analysis

All statistical tests were performed using the software SPSS 20.0 (SPSS, Inc., Chicago, IL, USA). Repeated measurement variables were analyzed using a mixed-effects model with a Bonferroni-corrected significance level used for pairwise comparison. The Mann–Whitney U test was used to compare the number of glaucoma medications before and after surgery. Nominal variables were analyzed using Pearson chi-squared test or Fisher’s exact test. The Kaplan–Meier curve representsed the success rate of surgery. Alpha values of 0.05 were used for statistical calculations.

## Results

Demographics and baseline characteristics of the eyes are presented in Table 1. A total of 35 patients (21with juvenile-onset open-angle glaucoma and 14 with POAG; 44 eyes) were included in this study, of whom 28 were male and 7 were female. The median age at the time of GATT was 38 years (range, 18 to 60). The mean preoperative IOP was 27.4 ± 8.8 mm Hg on 3.6 ± 0.7 medications. All except one case had moderate or severe damage. Three eyes were pseudophakic. Thirty-five eyes (79.5%) had one prior incisional glaucoma surgery (28 with a prior trabeculectomy, 6 with Ex-press shunt implantation, and one with canaloplasty), and 9 eyes had two prior incisional glaucoma surgeries (6 with repeat trabeculectomy, one with trabeculectomy and Ex-Press shunt implantation, one with trabeculectomy and Ahmed valve implantation, and one with trabeculectomy and endoscopic cyclophotocoagulation). Three patients underwent prior trabeculectomy and selective laser trabeculoplasty. Preoperative gonioscopy revealed open angles in all eyes.

**Table 1:** Demographics and ocular characteristics of subjects

Variables	
No. patients	35
Glaucoma type	
POAG	14
JOAG	21
No. eyes	44
Sex, male/female	28/7
Age, median (mean) (y)	38 (36.5)
Range (y)	18–60
Prior incisional glaucoma surgeries	
Eyes with 1 prior incision surgery, n (%)	35 (79.5)
Trab, n	28
Ex-press, n	6
Canaloplasty, n	1
Eyes with 2 prior incision surgeries, n (%)	9 (20.5)
2 Trab, n	6
1 Trab + 1 Ex-Press, n	1
1 Trab + 1 Ahmed valve, n	1
1 Trab + 1 ECP, n	1
Eyes with prior SLT, n	3
Pseudophakia, n	3
Glaucoma staging	
Mild, n (%)	1 (2.3)
Moderate, n (%)	7 (15.9)
Severe, n (%)	36 (81.8)
Preoperative IOP, mean ± SD (mm Hg)	27.4 ± 8.8
Preoperative glaucoma medications, mean ± SD	3.6 ± 0.7

*POAG* Primary open-angle glaucoma, *JOAG* Juvenile-onset open-angle glaucoma, *Trab* Trabeculectomy, *Ex-Press* Ex-Press shunt implantation, *ECP* Endoscopic cyclophotocoagulation, *SLT* Selective laser trabeculoplasty, *SD* Standard deviation

**Fig. 1:**
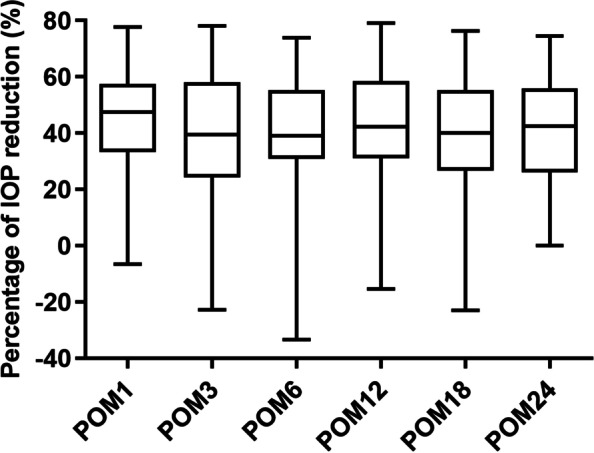
Box-and-whisker plot showing postoperative changes of intraocular pressure (IOP) at each visit. The median (50th percentile) is represented by the horizontal centerline, and the 25th and 75th percentiles as the lower and upper limits of the box. The upper and lower horizontal bars represent the maximum and minimum values, respectively, of a 1.5 interquartile range. POM, postoperative month

**Fig. 2:**
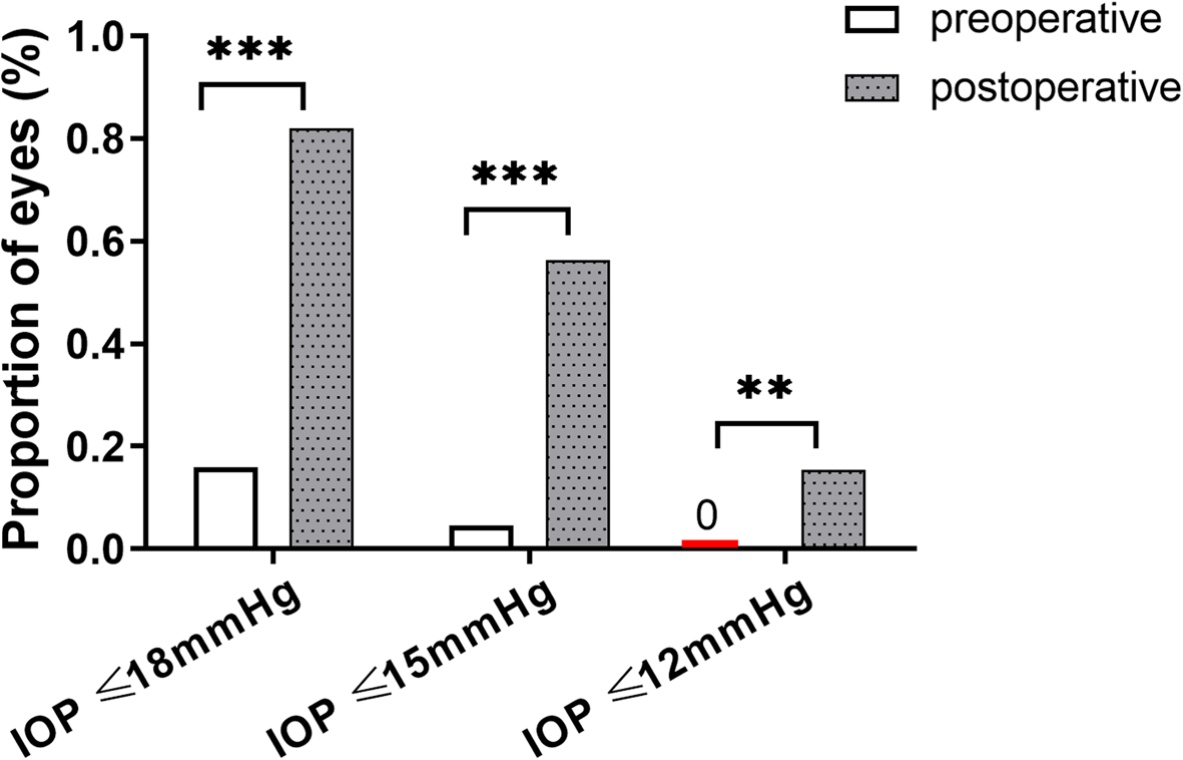
Percentages of eyes with IOP ≤ 18, ≤ 15, and ≤ 12 mm Hg preoperatively and at the 24-month visit after gonioscopy-assisted transluminal trabeculotomy. ** *P* < 0.01,*** *P* < 0.001

**Fig. 3:**
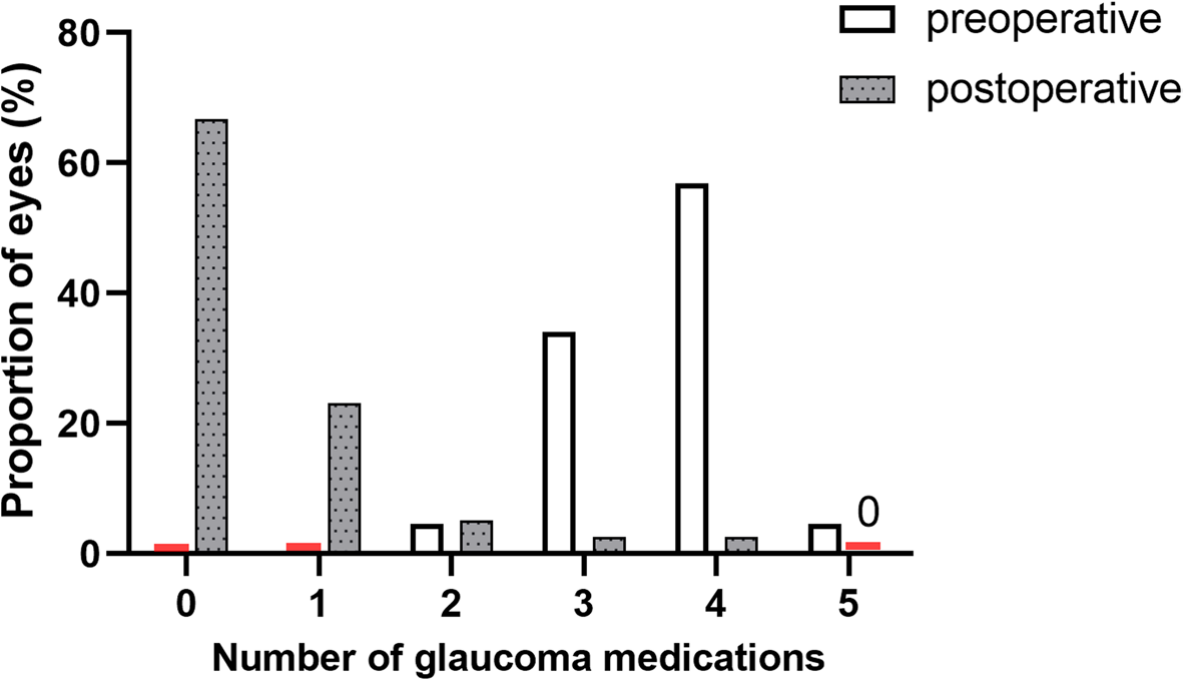
Proportions of eyes on 0, 1, 2, 3, 4, and 5 glaucoma medications preoperatively and at the 24-month visit after gonioscopy-assisted transluminal trabeculotomy

**Fig. 4:**
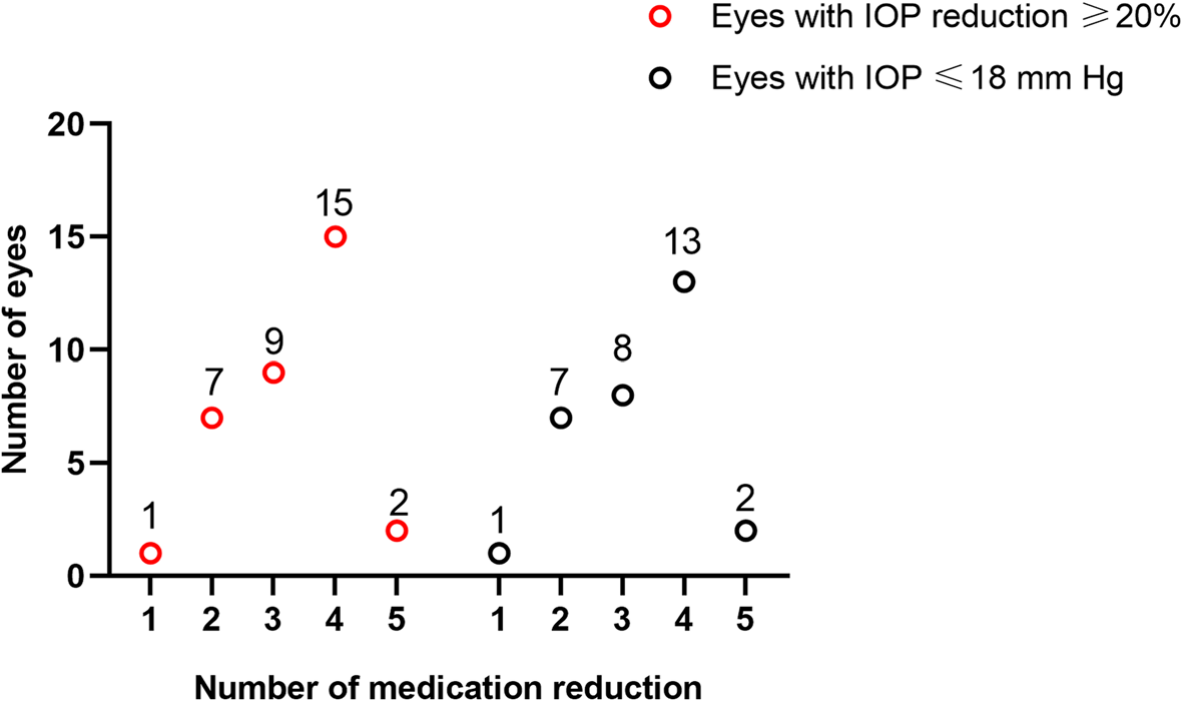
Numbers of eyes with intraocular pressure (IOP) reduction ≥ 20% on fewer glaucoma medications (red circles) and eyes with IOP ≤ 18 mm Hg on fewer glaucoma medications (black circles) 24 months after gonioscopy-assisted transluminal trabeculotomy

At 1, 3, 6, 12, 18, and 24 months after surgery, the mean IOP and the number of glaucoma medications were lower than that before surgery (all *P* < 0.001) (Table 2). Mean IOP decreased from (27.4 ± 8.8) mm Hg preoperatively to (15.3 ± 2.7) mm Hg 24 months after surgery, with the number of glaucoma medications decreasing from (3.6 ± 0.7) to (0.5 ± 0.9). At each follow-up visit, more than 75% of eyes reached IOP reduction greater than 20% (Fig. 1). At the 24-month visit, 82.1% of the eyes had IOP ≤ 18 mm Hg (versus 15.9% preoperatively, *P* < 0.001), 56.4% reached IOP ≤ 15 mm Hg (versus 4.6% preoperatively, *P* < 0.001), and 15.4% achieved IOP ≤ 12 mm Hg (compared to none preoperatively, *P* = 0.009) (Fig. 2). While 95.5% of eyes took 3 or more medications preoperatively, 66.7% did not take glaucoma medication 24 months after GATT (Fig. 3). Figure 4 showed 34 eyes (77.3%) achieved IOP reduction greater than 20% on fewer medications; 31 eyes (70.5%) had IOP ≤ 18 mm Hg on fewer medications.

**Table 2:** IOP and number of glaucoma medications at baseline and over 2-year follow-up

Variables	No. eyes	IOP (mm Hg)	Glaucoma medications
Preoperative	44	27.4 ± 8.8	3.6 ± 0.7
Postoperative			
1 month	44	14.2 ± 3.8	0.8 ± 1.2
3 months	44	15.5 ± 4.2	0.5 ± 0.9
6 months	42	15.0 ± 3.4	0.4 ± 0.8
12 months	40	14.7 ± 2.8	0.4 ± 0.8
18 months	40	15.5 ± 2.5	0.5 ± 1.0
24 months	39	15.3 ± 2.7	0.5 ± 0.9
*P* value		< 0.001	< 0.001

The IOP and number of anti-glaucoma medications are expressed as mean ± standard deviations

*IOP* Intraocular pressure

**Fig. 5:**
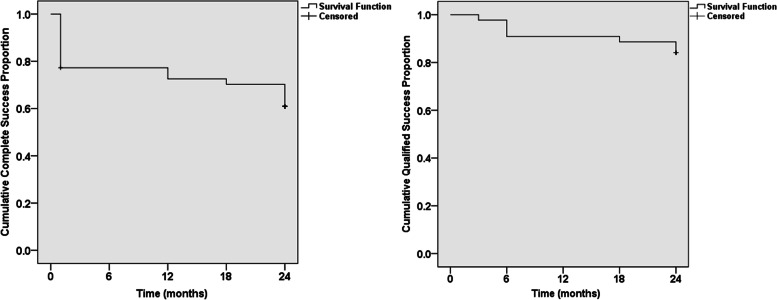
The cumulative probability of complete (**A**) and qualified (**B**) success rate

Figure 5 showed the Kaplan–Meier survival plots. At 24 months postoperatively, the complete and qualified success rate were 60.9% and 84.1%, respectively. Table 3 demonstrated the efficacy outcomes of GATT for patients with different types of previous surgeries. Five eyes with uncontrolled IOP after GATT underwent repeated surgery (three eyes underwent repeated trabeculectomy, one underwent goniosynechialysis, and one underwent CO2 laser-assisted sclerectomy surgery).

**Table 3:** Outcomes of GATT for eyes with different types of previous surgeries

Types of prior glaucoma surgeries	No. eyes	Complete success rate	Qualified success rate
1 Trab	28	53.6%	82.1%
1 Ex-Press	6	100%	100%
1 Canaloplasty	1	0	0
2 Trab	6	83.3%	100%
1 Trab + 1 Ex-Press	1	0	0
1 Trab + 1 Ahmed valve	1	0	100%
1 Trab + 1 ECP	1	100%	100%

*Trab* Trabeculectomy, *Ex-Press* Ex-Press shunt implantation, *ECP* Endoscopic cyclophotocoagulation

Hyphema occurred in all subjects, which resolved within 1 to 2 weeks after surgery. IOP spike (an IOP of ≥ 30 mmHg within one month postoperatively) occurred in 19 eyes (16 eyes in the first week after operation and 3 eyes in the second week). IOP spike was treated by glaucoma medications or by releasing aqueous humor from the paracentesis site. In this case series, 11 eyes with IOP spike and 6 eyes without IOP spike did not meet the definition of complete success (11/19 vs. 6/25, *P* = 0.022). Ciliochoroidal detachment was identified in 4 eyes by anterior-segment OCT imaging, which resolved simultaneously within the first month after surgery.

## Discussion

A prior failed filtering procedure is one known risk factor for bleb failure [24–26]. This study evaluated the efficacy and safety of GATT in treating patients with OAG and previous failed filtering surgeries. Patients recruited in this study were much younger than these in similar previous studies [20]. Our results showed a significant reduction of IOP and glaucoma medication after GATT. The qualified success rate was close to 80% at 24 months after surgery.

The current GATT study present a high rate-of-success as compared to previous reports. Cubuk MO et al. [22] demonstrated the limited efficacy of a 5–0 prolene-suture GATT for POAG with failed trabeculectomy, while Grover DS et al. [20] reported that the mean IOP decrease was 9.4 mm Hg with a 0.8 decrease in glaucoma medications after GATT for open-angle glaucoma with prior incisional glaucoma surgery. Our study demonstrated a larger decrease in both mean IOP and glaucoma medications after surgery. This may partly be due to the following points. First, the average age of our study was younger than that in prior studies [20, 22]. Outflow resistance may be more localized to the trabecular meshwork among younger patients, and GATT targets dysfunctional trabecular meshwork. Shi Y et al. [27] reported that older age was one of the risk factors for GATT failure when treating juvenile open angle glaucoma. Salimi A et al. [28] also reported favorable results of GATT in treating younger to middle-aged adults with open angle glaucoma. Our results were consistent with these studies. Second, we used illuminated microcatheters while Cubuk MO and colleagues used 5–0 prolone sutures. Microcatheters can offer the advantage of visualization of the catheter tip location within the Schlemm’s canal and help with catheter placement.

Prior incisional glaucoma surgery does not hinder microcatheter cannulation in the current study, which was consistent with the results reported by Grover DS and his colleagues [20]. In contrast, there was higher rate of failure to catheterize the Schlemm’s canal in treating primary congenital glaucoma after failed glaucoma surgeries [19]. The discrepancy may partly be attributed to the anatomical difference in the anterior chamber angle between congenital versus other forms of glaucoma. Anterior chamber angle dysplasia or developmental anomaly of the Schlemm’s canal may be more severe in primary congenital glaucoma, which makes it harder to circumferentially catheterize the canal.

In addition to surgical suture and microcatheter, the TRAB360 device has also been used for 360-degree ab-interno trabeculotomy for patients with refractory glaucoma, 20% of which had prior incisional glaucoma surgery [21]. Unlike our study, secondary open-angle glaucoma, chronic angle-closure glaucoma, congenital glaucoma and neovascular glaucoma were also enrolled by Sarkisian SR and the colleagues [21]. In their study, the mean IOP was reduced from 23.7 ± 6.0 mmHg preoperatively to 15.7 ± 5.5 mmHg at the 12-month visit. The current study together with the work of Grover DR [20] and of Sarkisian SR [21] showed favorable results of GATT when treating refractory glaucoma, including cases with prior failed incisional glaucoma surgery. Moreover, our prior evaluation indicated that prior incisional glaucoma surgery is not a risk factor for GATT failure in treating juvenile open angle glaucoma [10].

Tube shunt procedures as well as repeat trabeculectomy are commonly used for patients with failed glaucoma surgery. In the TVT study, both tube shunt surgery and trabeculectomy with mitomycin C produced an IOP reduction of greater than 40% [29]. Nassiri N et al. [30] also evaluated the efficacy of trabeculectomy and tube shunt surgery in treating patients with failed filtering surgery and reported similar percentage of IOP reduction. The mean IOP at two years in both studies were slightly lower than that in this study; our results supported that GATT may be desirable for subjects with failed incisional glaucoma surgery who are at high risk for failures for repeated filtering surgery. Randomized controlled studies are warranted to address whether GATT is comparable to traditional filtering surgery in treating these refractory cases.

GATT showed a favorable safety profile for refractory glaucoma in our study and published literature [20, 22]. While more than 20% of patients experienced serious complications associated with reoperation and/or vision loss of 2 or more lines in TVT study [29], there was no sight-threatening event in the current study and re-operation was performed for five eyes to control IOP. IOP spikes following GATT are drawing more attention. Salimi A and colleagues [28] reported that IOP spike was not related to surgical failure, while our results and other studies indicated postoperative IOP spike may be associated with surgical failure [5, 27]. Shi et al. [27] indicated the median IOP spike duration was 3.5 days (range 1–21) after GATT; notably, prolonged IOP spike may be a predictor of GATT failure. The cause of IOP spike still remains unclear. Previous studies showed eyes receiving corticosteroids [11] or those with advanced glaucoma [27] were more likely to suffer IOP spike.

The current study is limited by the small sample size and the follow-up period of 24 months. Additional study with larger sample sizes and longer follow-up periods are necessary to evaluate its long-term effects. For several patients, both eyes were included in this study, which may affect interpretation of the results. IOP spike is a common postoperative complications and attracts attention. Since patients in this study are not followed daily until the end of the spike, we cannot show the exact duration of IOP spike. Further studies are needed to investigate this issue. The limitations universal to retrospective studies such as non-randomization with no control group may also affect the results. However, it does provide real-world data on the safety and efficacy of GATT in treating refractory glaucoma.

## Conclusions

GATT is safe and effective for the treatment of OAG patients who had prior failed incisional glaucoma surgery. Randomized controlled studies with long-term follow-up should be done to better understand the efficacy of GATT.

## Data Availability

All data included in this study are available from the corresponding author on reasonable request.

## Abbreviations

POAG: Primary open-angle glaucoma
GATT: Gonioscopy-assisted transluminal trabeculotomy
IOP: Intraocular pressure

## References

[1] Tham YC, Li X, Wong TY, Quigley HA, Aung T, Cheng CY (2014). Global prevalence of glaucoma and projections of glaucoma burden through 2040: a systematic review and meta-analysis. Ophthalmology.

[2] Song P, Wang J, Bucan K, Theodoratou E, Rudan I, Chan KY (2017). National and subnational prevalence and burden of glaucoma in China: A systematic analysis. J Glob Health.

[3] Qiao C, Zhang H, Cao K, Tian J, Chung TY, Shan J (2022). Changing Trends in Glaucoma Surgery Over the Past 5 Years in China. J Glaucoma.

[4] Grover DS, Godfrey DG, Smith O, Feuer WJ, Montes de Oca I, Fellman RL (2014). Gonioscopy-assisted transluminal trabeculotomy, ab interno trabeculotomy: technique report and preliminary results. Ophthalmology.

[5] Rahmatnejad K, Pruzan NL, Amanullah S, Shaukat BA, Resende AF, Waisbourd M (2017). Surgical Outcomes of Gonioscopy-assisted Transluminal Trabeculotomy (GATT) in Patients With Open-angle Glaucoma. J Glaucoma.

[6] Grover DS, Smith O, Fellman RL, Godfrey DG, Gupta A, Montes de Oca I (2018). Gonioscopy Assisted Transluminal Trabeculotomy: An Ab Interno Circumferential Trabeculotomy - 24-month Follow-up. J Glaucoma.

[7] Aktas Z, Ucgul AY, Bektas C, Sahin KS (2019). Surgical Outcomes of Prolene Gonioscopy Assisted Transluminal Trabeculotomy in Patients with Moderate to Advanced Open Angle Glaucoma. J Glaucoma.

[8] Boese EA, Shah M (2019). Gonioscopy-assisted Transluminal Trabeculotomy (GATT) is An Effective Procedure for Steroid-induced Glaucoma. J Glaucoma.

[9] Smith BL, Ellyson AC, Kim WI (2018). Trabectome-Initiated Gonioscopy-Assisted Transluminal Trabeculotomy. Mil Med.

[10] Wang Y, Wang H, Han Y, Shi Y, Xin C, Yin P, et al. Outcomes of gonioscopy-assisted transluminal trabeculotomy in juvenile-onset primary open-angle glaucoma. Eye (Lond). 2020. 10.1038/s41433-020-01320-0 PMC8452612 33262477

[11] Chen J, Wang YE, Quan A, Grajewski A, Hodapp E, Vanner EA (2020). Risk Factors for Complications and Failure after Gonioscopy-Assisted Transluminal Trabeculotomy in a Young Cohort. Ophthalmol Glaucoma.

[12] Sato T, Kawaji T (2021). 12-month randomised trial of 360° and 180° Schlemm's canal incisions in suture trabeculotomy ab interno for open-angle glaucoma. Br J Ophthalmol.

[13] Sharkawi E, Lindegger DJ, Artes PH, Lehmann-Clarke L, El Wardani M, Misteli M (2021). Outcomes of gonioscopy-assisted transluminal trabeculotomy in pseudoexfoliative glaucoma: 24-month follow-up. Br J Ophthalmol.

[14] Grover DS, Smith O, Fellman RL, Godfrey DG, Butler MR, Montes de Oca I (2015). Gonioscopy assisted transluminal trabeculotomy: an ab interno circumferential trabeculotomy for the treatment of primary congenital glaucoma and juvenile open angle glaucoma. Br J Ophthalmol.

[15] Hopen ML, Gallardo MJ, Grover D (2019). Gonioscopy-assisted Transluminal Trabebeculotomy in a Pediatric Patient with Steroid-induced Glaucoma. J Glaucoma.

[16] Areaux RG, Grajewski AL, Balasubramaniam S, Brandt JD, Jun A, Edmunds B (2020). Trabeculotomy Ab Interno With the Trab360 Device for Childhood Glaucomas. Am J Ophthalmol.

[17] Lehmann-Clarke L, Sadeghi Y, Guarnieri A, Sharkawi E (2020). Gonioscopy-assisted transluminal trabeculotomy using an illuminated catheter for infantile primary congenital glaucoma Case series. Am J Ophthalmol Case Rep.

[18] Sachdev A, Khalili A, Choi J, Stead RE, Sung VCT (2020). Gonioscopy-assisted Transluminal Trabeculotomy in Uveitic Glaucoma Secondary to Juvenile Idiopathic Arthritis. J Glaucoma.

[19] Shi Y, Wang H, Oatts J, Cao K, Xin C, Liang X (2020). Ab interno vs ab externo microcatheter-assisted trabeculotomy for primary congenital glaucoma with clear cornea. Clin Exp Ophthalmol.

[20] Grover DS, Godfrey DG, Smith O, Shi W, Feuer WJ, Fellman RL (2017). Outcomes of Gonioscopy-assisted Transluminal Trabeculotomy (GATT) in Eyes With Prior Incisional Glaucoma Surgery. J Glaucoma.

[21] Sarkisian SR, Mathews B, Ding K, Patel A, Nicek Z (2019). 360 degrees ab-interno trabeculotomy in refractory primary open-angle glaucoma. Clin Ophthalmol.

[22] Cubuk MO, Ucgul AY, Unsal E (2020). Gonioscopy-assisted transluminal trabeculotomy as an option after failed trabeculectomy. Int Ophthalmol.

[23] Hodapp EPRI, Anderson DR (1993). Clinical Decisions in Glaucoma.

[24] Law SK, Shih K, Tran DH, Coleman AL, Caprioli J (2009). Long-term outcomes of repeat vs initial trabeculectomy in open-angle glaucoma. Am J Ophthalmol.

[25] Broadway DC, Chang LP (2001). Trabeculectomy, risk factors for failure and the preoperative state of the conjunctiva. J Glaucoma.

[26] Sugimoto Y, Mochizuki H, Ohkubo S, Higashide T, Sugiyama K, Kiuchi Y (2015). Intraocular Pressure Outcomes and Risk Factors for Failure in the Collaborative Bleb-Related Infection Incidence and Treatment Study. Ophthalmology.

[27] Shi Y, Wang H, Oatts JT, Xin C, Yin P, Zhang L (2021). A prospective study of intraocular pressure spike and failure after gonioscopy-assisted transluminal trabeculotomy in juvenile open angle glaucoma. Am J Ophthalmol.

[28] Salimi A, Nithianandan H, Al Farsi H, Harasymowycz P, Saheb H (2021). Gonioscopy-Assisted Transluminal Trabeculotomy in Younger to Middle-Aged Adults: One-Year Outcomes. Ophthalmol Glaucoma.

[29] Gedde SJ, Schiffman JC, Feuer WJ, Herndon LW, Brandt JD, Budenz DL (2009). Three-Year Follow-up of the Tube Versus Trabeculectomy Study. Am J Ophthalmol.

[30] Nassiri N, Syeda S, Tokko H, Thipparthi M, Cohen MI, Kim C (2020). Three-year outcomes of trabeculectomy and Ahmed valve implant in patients with prior failed filtering surgeries. Int Ophthalmol.

